# 4.1: Nonbiopsy of presumed fibroadenomas in patients <30 years: is it safe? A single unit experience and review of European practice

**DOI:** 10.1186/bcr3498

**Published:** 2013-11-08

**Authors:** K Taylor, H Vandersluis, P Britton, M Wallis

**Affiliations:** 1 Cambridge Breast Unit, Cambridge University Hospitals NHS Foundation Trust, Cambridge, UK

## Introduction

Previous research in this centre enabled the introduction of a local protocol of nonbiopsy and discharge of women <30 years old with presumed fibroadenomas (FAs). Four years on, we have audited protocol accuracy, effect on biopsy workload, safety and its potential for influencing UK and European practice.

## Methods

Women aged <30 attending the breast unit between 1 February 2009 and 31 January 2013 were retrospectively identified. Clinical and imaging results were scored following national guidelines locally adapted to incorporate a nonbiopsy protocol such that presumed FAs scored E2/U2 meeting specific protocol criteria were discharged without core biopsy. In addition, a survey assessing European practice was completed by 31 centres.

## Results

A total of 1,571 women age <30 were referred to the breast unit. Seven cancers were diagnosed, all aged 25 to 29. In total, 266 presumed FAs E2/U2, meeting nonbiopsy criteria were discharged without biopsy, 84 were aged 25 to 29. Fourteen re-attended with increase in size, none biopsied but five excised due to patient choice. Sixty-six E2/U2, probable FAs were biopsied due to noncompliance with the protocol. Of these, 54 were FAs, seven phyllodes tumours, and one cancer. The protocol resulted in a 78% reduction in biopsy workload in women aged ≥20 and a 72% reduction in women aged 25 to 29. No cancers developed in discharged patients, mean follow up 2.4 years. European survey results demonstrate 29% of respondents routinely sample FAs in women age ≥20, 55% in women age ≥25. Seventy-nine per cent of non-UK respondents follow-up FAs regardless of patient age.

## Conclusion

With rigorous adherence, our nonbiopsy protocol for presumed FAs in women age <30 appears safe and reduces biopsy/follow-up workload.

